# Virological response and safety of 24-week telaprevir alone in Japanese patients infected with hepatitis C virus subtype 1b

**DOI:** 10.1111/j.1365-2893.2012.01640.x

**Published:** 2013-03

**Authors:** J Toyota, I Ozeki, Y Karino, Y Asahina, N Izumi, S Takahashi, Y Kawakami, K Chayama, N Kamiya, K Aoki, I Yamada, Y Suzuki, F Suzuki, H Kumada

**Affiliations:** 1 Department of Gastroenterology, Sapporo Kosei General HospitalHokkaido, Japan; 2 Division of Gastroenterology and Hepatology, Musashino Red Cross HospitalTokyo, Japan; 3 Division of Frontier Medical Science, Department of Medical and Molecular Science, Programs for Biomedical Research, Graduate School of Biomedical Science, Hiroshima UniversityHiroshima, Japan; 4 Research and Development Division, Mitsubishi Tanabe Pharma CorporationTokyo, Japan; 5 Department of Hepatology, Toranomon HospitalTokyo, Japan

**Keywords:** hepatitis C virus, monotherapy, subtype 1b, telaprevir

## Abstract

Hepatitis C virus (HCV) subtype 1b, which infects approximately 70% of Japanese carriers, is likely to be more eradicable by a telaprevir regimen than subtype 1a because of the higher genetic barrier of Val^36^ and Arg^155^ substitutions. The aims of this exploratory study were to evaluate the virological response and safety of 24-week oral administration of telaprevir alone in chronic HCV subtype 1b infection. Fifteen treatment-naïve patients were treated with telaprevir 750 mg every 8 h for 24 weeks. All patients were Japanese whose median age was 58.0 years (range: 45–68), and six patients (40%) were men. Median baseline HCV RNA level was 6.80 log_10_ IU/mL (range: 3.55–7.10). The HCV RNA levels decreased to undetectable in five patients (33%) within 8 weeks. Three patients (20%) with negative HCV RNA by Week 4 achieved end of treatment response. One patient (7%) who achieved sustained virological response had a low baseline viraemia of 3.55 log_10_ IU/mL. Most of the adverse events including anaemia and skin disorders were mild to moderate. Developed variants were T54A and A156V/T/F/Y with or without secondary substitutions rather than V36M ± R155K. Telaprevir alone for 24 weeks in Japanese patients with HCV subtype 1b resulted in an sustained viral response rate of 7% (1/15) and was well tolerated for 24 weeks. These results will support the implementation of further studies on oral combination of telaprevir with other direct-acting antiviral agents in patients infected with HCV subtype 1b.

## Introduction

The World Health Organization (WHO) estimates that approximately 170 million people are infected with hepatitis C virus (HCV) [1]. In Japan, it is estimated that more than 1.5 million people are chronically infected with hepatitis C.

Telaprevir is a novel peptidemimetic HCV NS3-4A protease inhibitor. The mechanism of inhibition involves the formation of a stable, reversible, covalent bond between the ketocarbonyl of telaprevir and the active site serine of NS3 protease. Recently, telaprevir was approved for patients with HCV genotype 1 infection in the United States (US), Canada, European Union (EU) and Japan. The Phase 3 studies showed that patients who received telaprevir in combination with pegylated interferon (PEG-IFN) and ribavirin (RBV) achieved significantly higher rates of sustained viral response (SVR) compared to those who received PEG-IFN and RBV alone, regardless of their prior treatment experience [2–4]. The Japanese Phase 3 studies of the telaprevir-based triple regimen also showed high SVR rates [5,6]. The most common side effects in the telaprevir-based triple regimen were anaemia, rash and IFN-induced systemic symptoms.

The epidemiology of HCV in Japan takes on a different aspect from US and EU; that is, the majority of patients are aged more than 55 years [7]. Accordingly, the RBV dose reduction rate and the frequency of discontinuation of telaprevir treatment in Japan are higher than those in US and EU [2–6]. Taking such problems with telaprevir in combination with PEG-IFN and RBV into consideration, IFN-free regimens may become very useful options and satisfy important unmet medical needs especially for intolerant patients with IFN-based regimens. Clinical trials of IFN-free therapy for patients with chronic hepatitis C would provide us with meaningful knowledge for the future development of HCV therapy. Interestingly, HCV subtype 1b, which infects approximately 70% of Japanese HCV carriers [8], is likely to be more eradicable by telaprevir regimens than subtype 1a because of the higher genetic barrier of Val^36^ and Arg^155^ substitutions [9,10]. When treating with direct-acting antiviral agent (DAA), HCV subtypes of genotype 1 are now an important factor that affects treatment response. The main aim of this exploratory study is to evaluate the virological response and safety of telaprevir as monotherapy for 24 weeks in Japanese patients infected with HCV subtype 1b.

## Patients and Methods

### Study design and organization

This Phase 2, single-arm, open-label study was conducted from January 2008 to February 2009 at Sapporo Kosei General Hospital, Musashino Red Cross Hospital, Toranomon Hospital and Hiroshima University Hospital. The study was conducted in accordance with the Declaration of Helsinki and Good Clinical Practices. Before starting the study, the protocol and informed consent forms were reviewed and approved by the institutional review board in each site. All patients provided written informed consent following sufficient explanation before participating in the study. All the patients received 750 mg telaprevir orally every 8 h (q8h) (2250 mg/day) after a meal for 24 weeks. Telaprevir was given as a 250-mg tablet. This study is registered in ClinicalTrials.gov NCT 00621296.

### Patients

Participants enrolled in this study were treatment-naïve, male or female chronic hepatitis C patients with the characteristics shown in Table 1 who met the inclusion criteria and did not conflict the exclusion criteria described previously [11], except the age and HCV RNA levels at the time of enrolment; age from 20 to 70 years and HCV RNA levels were not defined.

**Table 1:** Patient characteristics, treatment duration and viral response

	Sex	Age	BMI (kg/m^2^)	Baseline HCV RNA (log_10_ IU/mL)	Treatment duration (day)	HCV RNA Nadir (log_10_ IU/mL)	Virological response
1	M	67	25.2	5.85	169 (complete)	Undetectable	Relapse
2	M	59	24.5	3.55	169 (complete)	Undetectable	SVR
3	F	45	18.7	6.80	44^*^	2.8	Breakthrough
4	F	68	20.9	7.05	43^†^	<1.2 detectable	Partial responder
5	F	48	21.5	6.45	169 (complete)	Undetectable	Breakthrough
6	F	57	20.9	4.75	43^*^	1.8	Breakthrough
7	F	51	19.9	5.95	170 (complete)	Undetectable	Partial responder
8	F	58	19.2	6.85	105^*^	1.5	Breakthrough
9	M	62	20.4	6.25	14^†^	1.4	Partial responder
10	M	58	24.5	7.10	39^*^	3.1	Breakthrough
11	M	63	16.2	7.00	74^*^	<1.2 detectable	Breakthrough
12	F	53	25.0	7.10	169 (complete)	Undetectable	Relapse
13	F	60	19.7	5.00	10^‡^	<1.2 detectable	Breakthrough
14	F	55	23.8	6.95	78^*^	<1.2 detectable	Breakthrough
15	M	50	27.5	6.90	26^‡^	1.3	Partial responder

HCV, Hepatitis C virus; SVR, sustained viral response. Subjects discontinued telaprevir because of ^*^viral breakthrough, ^†^AE and ^‡^other reasons.

### Virological responses

Virological response to telaprevir was evaluated based on the HCV RNA kinetics in patients. Serum HCV RNA levels were measured using the COBAS TaqMan HCV test (Roche Diagnostics Co., Ltd., Tokyo, Japan). The linear dynamic range was 1.2–7.8 log_10_ IU/mL. A qualitative result below the lower limit of quantification (LOQ) was also determined as positive (1.0) and negative (0.5). Measurements were obtained on Week 4 before the first dose, Days 1 (prior to the first dosing) and 3, Weeks 1, 2, 4, 6, 8, 10, 12, 14, 16, 18, 20, 22, and 24 of the treatment period, and Weeks 2, 4, 8, 12, 16, 20, and 24 of the follow-up period. Day 1 was defined as the date of starting telaprevir treatment.

Sustained viral response was defined as an undetectable HCV RNA level at 24 weeks after the end of treatment. Relapse was defined as the reappearance of serum HCV RNA during the follow-up period from the state of undetectable serum HCV RNA at the end of treatment. Breakthrough was defined as the state when the viral level increased by 2 log_10_ IU/mL from nadir or a level of more than 3 log_10_ IU/mL after reaching undetectable levels during treatment. Partial responders were subjects whose HCV RNA level dropped by at least 2 log_10_ IU/mL during treatment but was still detected at the end of treatment.

### Sequence analysis at HCV NS3 protease domain

HCV RNA was isolated from serum samples collected on the same day for the measurement of HCV RNA levels. A DNA fragment of 543 bases long (181 amino acids) from the NS3 protease domain was amplified by nested RT-PCR and cloned. At least 39 clones per specimen were sequenced bi-directionally. The limit of detection for the sequencing analysis was 3.0 log_10_ IU/mL.

### Safety assessments

Safety of telaprevir was assessed by clinical laboratory tests, vital signs, abdominal ultrasonography and AEs. Twelve-lead electrocardiogram (ECG) examinations were performed once during the screening period. These safety parameters were reported at regular intervals from 4 weeks before the first dosing to the end of the follow-up period.

### Statistical analysis

Statistical analyses were performed using the statistical software SAS Version 9.1.3 (SAS Institute Inc., Cary, NC, USA). Reported AEs were classified according to MedDRA/J version 12.0 (MedDRA Japanese Maintenance Organization, Tokyo, Japan).

## Results

### Baseline characteristics

Fifteen treatment-naïve patients infected with HCV subtype 1b were enrolled in this study. Baseline characteristics of patients are shown in Table 1. All patients were Japanese whose median age was 58.0 years (range: 45–68); 6 (40.0%) patients were men. Patients over 54 years of age accounted for 66.7% (10 of 15). Median baseline HCV RNA level was 6.80 log_10_ IU/mL (range: 3.55–7.10). The median BMI was 20.9 kg/m^2^ (range: 16.2–27.5).

### Virological response

**Fig. 1:**
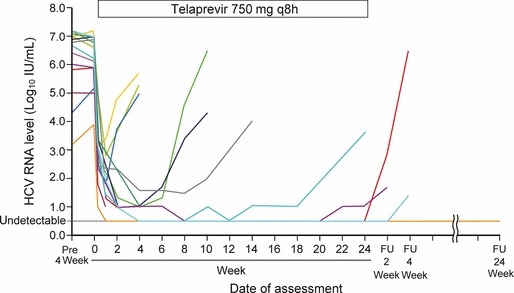
HCV RNA kinetics during and after treatment with telaprevir monotherapy.

Telaprevir alone caused a rapid decrease in HCV RNA levels after the initiation of treatment in all patients. The average changes were −3.24 log_10_ IU/mL on Day 3 and −4.24 log_10_ IU/mL on Week 1 (Fig. 1). The average of maximum reduction in each patient was 5.01 log_10_ IU/mL. The HCV RNA levels became undetectable in 1, 3, 3 and 5 patients at Weeks 1, 4, 6 and 8, respectively. Three patients with negative HCV RNA after 4 weeks achieved end of treatment response (ETR), of whom one patient achieved a SVR. The patient who achieved SVR had the lowest baseline viral load (3.55 log_10_ IU/mL) among all the patients.

Ten of 15 patients discontinued the telaprevir treatment because of the following reasons: six patients because of viral breakthrough, two patients because of AEs, one patient because of own drug discontinuation and one patient who met the exclusion criteria after administration.

### Safety

AEs observed in two or more patients in this study are shown in Table 2. During the study, 14 of 15 patients experienced 80 AEs in total and 62 events were judged as adverse drug reactions. The common AEs that occurred in more than 25% of patients were rash (53.5%), anaemia (46.7%), low-density lipoprotein (LDL) increases (40.0%), blood uric acid increase (26.7%) and pruritus (26.7%). Two patients discontinued telaprevir treatment because of AEs (herpes zoster or rash pruritic). Except for the herpes zoster whose severity was judged as severe and serious, all the events were mild to moderate. Fifty of the 80 AEs were observed within the first 4 weeks.

**Table 2:** Incidence of adverse events that occurred in two or more patients

	*N* = 15			
	
	Mild n (%)	Moderate n (%)	Severe n (%)	Total n (%)
Rash	5 (33.3)	3 (20.0)	0 (0.0)	8 (53.3)
Anaemia	7 (46.7)	0 (0.0)	0 (0.0)	7 (46.7)
Low-density lipoprotein increased	6 (40.0)	0 (0.0)	0 (0.0)	6 (40.0)
Blood uric acid increased	4 (26.7)	0 (0.0)	0 (0.0)	4 (26.7)
Pruritus	3 (20.0)	1 (6.7)	0 (0.0)	4 (26.7)
Anorexia	3 (20.0)	0 (0.0)	0 (0.0)	3 (20.0)
Dysgeusia	3 (20.0)	0 (0.0)	0 (0.0)	3 (20.0)
Headache	3 (20.0)	0 (0.0)	0 (0.0)	3 (20.0)
Diarrhoea	2 (13.3)	0 (0.0)	0 (0.0)	2 (13.3)
Pyrexia	2 (13.3)	0 (0.0)	0 (0.0)	2 (13.3)
Thirst	2 (13.3)	0 (0.0)	0 (0.0)	2 (13.3)
Nasopharyngitis	2 (13.3)	0 (0.0)	0 (0.0)	2 (13.3)
Blood creatinine increased	2 (13.3)	0 (0.0)	0 (0.0)	2 (13.3)
Blood triglycerides increased	2 (13.3)	0 (0.0)	0 (0.0)	2 (13.3)
Platelet count decreased	2 (13.3)	0 (0.0)	0 (0.0)	2 (13.3)
Dizziness	1 (6.7)	1 (6.7)	0 (0.0)	2 (13.3)

MedDRA (Ver.12.0).

**Fig. 2:**
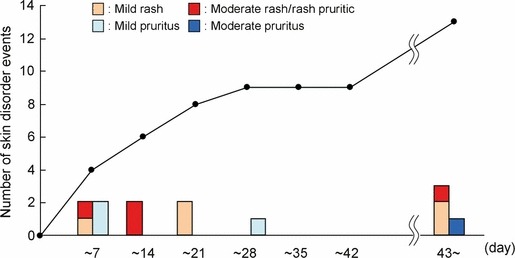
Rash and pruritus occurrence.

In relation to skin AEs, rash, pruritus and rash pruritic were observed in 8, 4 and 1 patients, respectively. The onset day of these events is described in Fig. 2. The range of the onset day was Day 1 to Day 113, and the median was Day 15. Rash in three patients, pruritus in one patient and rash pruritic in one patient were moderate, and the others were mild. One patient discontinued telaprevir at Week 6 because of moderate rash pruritic. Most of the skin AEs were treated with oral antihistamines or topical steroids.

**Fig. 3:**
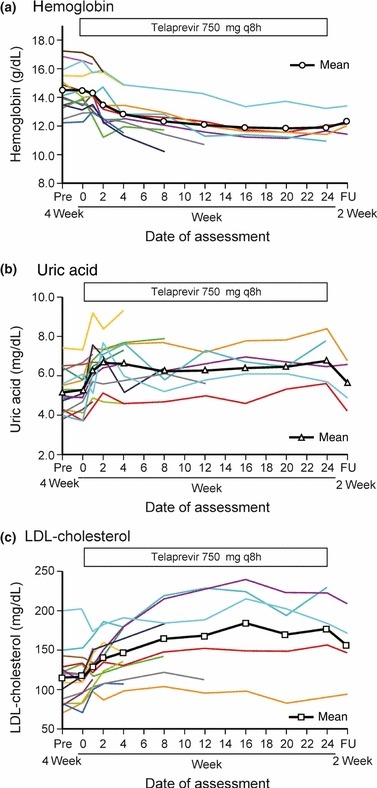
Changes in (a) hemoglobin, (b) uric acid, (c) LDL-cholesterol.

A decrease in haemoglobin levels was observed in all patients (Fig. 3a). Seven of 15 patients developed anaemia during and after the treatment. All anaemia events were mild and no patient needed discontinuation of telaprevir. Uric acid and LDL cholesterol increased during the treatment (Fig. 3b,c), but these changes were mild and no patient needed any medication for these AEs. There were no substantial increases in levels of alanine aminotransferase (ALT), aspartate aminotransferase (AST) and total bilirubin (T-bil).

### Sequence analysis at HCV NS3 protease domain

**Fig. 4:**
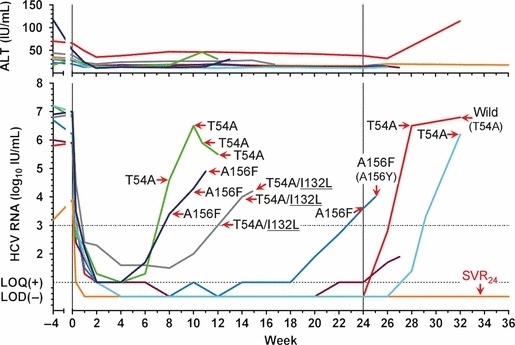
Viral kinetics and predominant variants during and after telaprevir monotherapy beyond 8 weeks. Besides predominant clones, minority clones which account for 10% and more in a specimen are also summarized by brace notation. Putative secondary resistant-associated mutation is indicated by underline.

Amino acid substitutions in the NS3 protease domain were examined in 39 clones or more in each sample. Before Week 8, V36A/G, T54A and A156T/V as single substitutions, and T54A + R155K and A156T/V + V158I as multiple substitutions were observed. Among two patients who discontinued telaprevir within 2 weeks, all clones but three in one patient were wild-type variants after withdrawal of telaprevir. In three patients who discontinued at Weeks 5–7 because of viral breakthrough, predominant clones possessed A156V/T substitutions after the nadir of viral load. Predominant variants observed during and after telaprevir monotherapy in the eight patients who received telaprevir beyond 8 weeks are shown in Fig. 4 together with HCV RNA levels. In the two patients who showed the lowest HCV RNA level of on Week 4, the predominant clones detected after viral breakthrough were A156F and T54A. One other patient with nadir HCV RNA level on Week 8 had a predominant clone of T54A + I132L after viral breakthrough. Among the five patients who completed the telaprevir treatment for 24 weeks as scheduled, two patients were HCV RNA positive at the end of treatment. One of these two patients had an A156F substitution at the end of treatment, and a A156Y substitution was also detected on Week 1 of the follow-up period. In the two patients who relapsed during the follow-up period, the predominant clone was T54A which shifted to the wild-type variant in one patient.

## Discussion

Although higher SVR rates and shorter duration of treatment were achieved by telaprevir in combination with PEG-IFN and RBV in US, EU and Japan [2–6], the DAA combination regimens also increased the frequency and severity of side effects usually observed in the PEG-IFN and RBV therapy. As most patients in Japan are aged people, IFN-free regimens are in urgent need because these patients are intolerant to IFN-based therapies [12–14].

In this exploratory study, one of 15 patients on telaprevir monotherapy was able to achieve SVR. A low viral load of <4 log_10_ IU/mL in this patient probably contributed to the achievement of SVR, and Suzuki *et al.* [15] published this case report in detail. Although the SVR rate obtained in the study was not beneficial enough, the telaprevir monotherapy could decrease HCV RNA levels dramatically in all cases. The severity of skin-related AEs during telaprevir monotherapy was milder than those of cases developing in the co-administration with PEG-IFN and RBV [5,6,16–18]. All the events were mild to moderate and manageable with antihistamines or topical steroids. Similarly to the skin-related events, decreases in haemoglobin levels were mild, and the incidence of anaemia was 46.7%. As all the anaemia events were mild, there was no need for discontinuation of telaprevir or use of any medications. Severe skin rash and anaemia observed in the therapy with telaprevir in combination with PEG-IFN and RBV are probably ascribable to the synergistic effect of these three drugs. Although the mechanism of uric acid and LDL cholesterol elevation during treatment with telaprevir has been established, these changes disappeared at the end of telaprevir dosing. Telaprevir was generally well tolerated in all the patients.

Amino acid substitutions in the HCV NS3 protease domain were monitored during the study. The relationship between these substitutions and resistance to NS3-4A protease inhibitors has been well documented by *in vitro*, *in vivo* and clinical studies [19–22]. In the eight patients who received the telaprevir monotherapy beyond 8 weeks, the predominant breakthrough variants were T54A and A156F, which were not observed at the earlier time points (Fig. 4). Furthermore, in the clones accounting for more than 10% of each specimen, the secondary substitution of V158I and I132L was identified along with the primary resistant-associated substitution of A156T/V and T54A, respectively, and a novel substitution of A156Y was also observed. This study confirms the higher genetic barrier of HCV subtype 1b against the V36M ± R155K substitutions. Our results clearly indicate that the prolonged telaprevir monotherapy leads to the development of various variants. As the replication fitness of drug-resistant variants tends to be lower than that of wild type, the former are likely to be overtaken by the wild-type virus under drug-free conditions within 3–7 months [11,23,24]. As Ozeki *et al.* [25] reported that four patients with favourable IL28B SNP who failed to eradicate HCV with telaprevir monotherapy were responsive to sequential therapy with PEG-IFN and RBV, the substitutions in the NS3 protease domain by the telaprevir treatment are not correlated with resistance to PEG-IFN and/or RBV directly as described previously [23,24]. Sequential therapy with PEG-IFN and RBV after relapse or viral breakthrough on telaprevir monotherapy might be a therapeutic option in some cases, including the case of low haemoglobin. By taking the error-prone nature of HCV replication into account, successful eradication with IFN-free DAA(s) regimens probably depends on how efficiently DAA can suppress various DAA-resistant variants that pre-exist and are selected under DAA pressure. The telaprevir-based combination therapy with other DAA(s) such as NS5A or NS5B polymerase inhibitors may be useful for successful treatment. Using a human chimeric liver mouse model for HCV infection, Ohara *et al.* [26] reported that the combination of telaprevir with a high-dose nucleoside analogue could successfully eradicate HCV infection. Recently, it was reported that the dual therapy with daclatasvir, an NS5A replication complex inhibitor, and asunaprevir, NS3-4A protease inhibitor, had high SVR rates in difficult-to-treat patients with subtype 1b and null responders [27,28]. These successful results are also helpful for us to consider telaprevir-based IFN-free regimens in combination with other DAAs against HCV.

In conclusion, telaprevir monotherapy was well tolerated and provided potent but temporary antiviral activity in Japanese patients with subtype 1b HCV, with an SVR rate of 7%. Most AEs were mild to moderate and much milder than those recorded in patients on combinations with PEG-IFN and RBV. As the essential characteristics of DAAs including telaprevir are substantially masked in the co-administration with other antivirals, the knowledge obtained from the long-term telaprevir monotherapy is most likely to contribute to the future HCV treatment with DAA-based regimens.

## Abbreviations

AE: adverse event
ALT: alanine aminotransferase
AST: aspartate aminotransferase
DAA: direct-acting antiviral agent
EU: European Union
HCV: Hepatitis C virus
LDL: low-density lipoprotein
LOQ: lower limit of quantification
PEG-IFN: pegylated interferon
RBV: ribavirin
SVR: sustained viral response
T-bil: total bilirubin
